# Latent class of multidimensional dependency in community-dwelling older adults: evidence from the longitudinal ageing study in India

**DOI:** 10.1186/s12877-024-04813-9

**Published:** 2024-02-28

**Authors:** Strong P. Marbaniang, Holendro Singh Chungkham

**Affiliations:** 1 St Anthony’s College, Department of Statistics, Shillong, Meghalaya India; 2 Division of Psychobiology and Epidemiology, Department of Psychology, Stockholm University, Stockholm, Sweden

**Keywords:** Latent class analysis, Multidimensional dependency, LASI, Aging, India

## Abstract

**Background:**

Existing studies have used ADL and IADL separately as measures of dependency. However, dependency is a heterogeneous and complex issue, and the dependency of each older adult is a synergistic combination of several functional activities. In this study, we assess the pattern of multidimensional dependency of older adults based on ADL, IADL, visual impairment, difficulty in climbing a flight of stairs, pushing or pulling objects, depressive symptoms, cognitive impairment, marital status, and economic distress. It is important to classify the dependency status of older adults because this will be key to evaluating the needs for care, and plan services that effectively cater for the needs of the older adults. The classification into different latent classes means that older adults within each class have the same needs of dependency but different needs between the latent classes. Our objective is to identify patterns of multidimensional dependency in older adults.

**Methods:**

Data from the Longitudinal Ageing Study in India (LASI) Wave-1, was used, the analytical sample consisted of 32,827 individuals of age 45 years and above. LCA was used to identify the multidimensional dependency class. LCA was conducted in *R* statistical package, using the poLCA package. The optimal number of classes was selected based on the comparison of model fit statistics. Independent variables were incorporated to explore the association between these variables and the latent class.

**Results:**

Based on nine indicator variables, three latent classes were identified: “Active Older adults”, “Moderately independent” and “Psychological and physically impaired”. The “Active older adults” profile is comprised of older adults who have a very low probability of needing help for any ADL, IADL and other activities. The “Moderately independent” class were characterized as those older adults who were visually impaired but less likely to need help for IADL activities. The “Psychological and physically impaired”, the smallest of all classes, comprised of older adults with poor dependency status.

**Conclusions:**

In this study, we found that the dependency status of older adults which is based on several domains of functional activity has been classified into three distinct classes. These three classes have distinct physical, psychological, economic, and socio-demographic characteristics in terms of activities in which help is required.

**Supplementary Information:**

The online version contains supplementary material available at 10.1186/s12877-024-04813-9.

## Background

As the 21^st^ century progresses the global population is growing older and is getting enlarged. Population ageing is a reflection of social, medical, and economic advancement and at the same time has a major socioeconomic consequence and puts major challenges to the healthcare system and other social entitlement programs. Projection suggests that the global population of 65 years and over will rise from 10% in 2022 to 16% in 2050, and the population of 80 years or over is projected to triple from 143 million in 2019 to 426 million in 2050 [1, 2]. With approximately 1.429 billion inhabitants, India has become the most populous country in the world surpassing China's population in 2023 [3]. In India, the population of older adults aged 65 years and over will increase from 90 million to 230 million between 2019 and 2050 [2]. The median age in India was 28 years in 2020 and was projected to increase to 38 years by 2050. This means that half of the population will be older than 38 years and the other half will be younger than 38 years of age in 2050 [4].

A growing ageing population has contributed to the rising prevalence of chronic diseases and disabilities and increasing demands on health and long-term social security systems. According to the Longitudinal Ageing Study in India (LASI) 2020, 27.7% of older adults of age 60 years and over have any chronic conditions [5]. Moreover, the disability rate was 51.8 per 1000 for the elderly and 84.1 per 1000 for 80 years and over, as compared to 22.1 per 1000 of the general population [6]. It is found that almost 24% and 48% of older adults in India are unable to perform at least one activity of daily living (ADL) and instrumental activities of daily living (IADL), respectively [5]. ADL is an index that evaluates the functional status as a measurement of an older adult's ability to perform basic activities of daily living independently. The index consists of six functions namely bathing, dressing, toileting, transferring, continence, and feeding. A score of 6 indicates total independence, 4 indicates moderate dependence, and 2 or less indicates total dependence [7]. ADL essentially indicates an individual’s ability to care for themselves without any assistance. Older adults who are unable to perform ADLs will have to depend on other individuals and/or mechanical devices. The functional ability assessment of older adults through ADL will help in determining whether an individual may require further assistance at home or a skilled nursing or long-term care facility may be a safer environment. It can help older adults or disabled person to determine their eligibility for availing assistance from the social help care programme [8]. Further, IADL is another index design to evaluate individual ability to live independently in a community. The major domains of IADL are the ability to make phone calls, shop, do laundry, cook, manage money, manage medication, and use transportation [9]. The assessment of IADL is commonly used in determining an individual’s needs for assistance and cognitive function [8].

While evaluating the dependency status of older adults, many studies have used ADL and IADL separately as measures of dependency [10–12]. However, dependency is a heterogeneous and complex issue, and the dependency of each older adult is a synergistic combination of several domains of functional activities [13, 14]. Therefore using only any single dependency measure to understand the complexity and heterogeneity of the dependency of older adults is not sufficient. Also, during the ageing process, older adults will experience a dependency transition, meaning that as age progresses, the dependency status of older adults will change from mild to severe dependency [15–17]. Hence, a better understanding of the diversity of functional activities may contribute to the knowledge about the heterogeneity of dependency. Also, it is evident from the previous studies that widowed is another important domain of dependency among elderly adults [18, 19]. It is found that widowhood significantly increases the likelihood of functional disability and economic distress among elderly adults compared to married couples with a spouse still alive [18]. It has been documented that low vision makes individuals more dependent as they face difficulty in working and to performing basic self-care activities [20]. Also, visually impaired older adults may be economically dependent on others to bear the health cost expenses [21]. Other factors resulting in older adults being more dependent are cognitive impairment and depression. Cognitive impairment and Depression lower individual quality of life and increase one’s dependency on others [22, 23]. In this study, we try to answer the question of whether there exists a latent classification on the dependency status of older adults, which allows investigating that will help to identify subgroups of older adults who need different levels of care. The evidence from many works of literature acknowledges that the dependency of older adults is linked with many factors. This study aimed to identify classes of multidimensional dependency based not only on functional limitation, but economic status, psychological, and social, and to determine the socio-demographic predictors of class membership. We hypothesised that dependency status is a multidimensional concept in older adults.

In this study, we used latent class analysis (LCA) to identify subgroups or classes of older adults based on the dependency variables. LCA is widely used in the fields of social, economic, psychological medicine, and health research, where the heterogeneous population is the focus of attention [24]. In geriatrics, LCA has been used to identify subgroups of social activities and health, depressive subtypes, and behavioural and psychological patterns in Alzheimer’s patients [25–27]. However, we find limited studies of its use to identify the pattern of multidimensional dependency in older adults. Latent class Analysis (LCA) is a person-centred analytical approach that allows the identification of homogeneous sub-groups in a heterogeneous population. A latent class analysis splits the population into mutually exclusive and exhaustive classes in such a way that people within the class have similar characteristics while people between the classes have different characteristics [28]. To explain the heterogeneity of dependency among older adults, we consider the physical, psychological, economic, and social domains of dependency. The observed indicators or the manifest variables used in LCA are physical (ADL, IADL, visual impairment, difficulty in climbing a flight of stairs, pushing or pulling objects), psychological dimension (depressive symptoms, cognitive impairment), social (marital status), and economic distress.

## Methods

### Research design

The data used in the analysis was drawn from the national representative Longitudinal Ageing Study in India (LASI) Wave-1, which was conducted across the country between April 2017 and December 2018. The LASI collects information on demographics, household economic status, chronic health conditions, symptom-based health conditions, functional health, mental health (cognitive and depression), biomarkers, work, health insurance and health utilisation, family and social network etc. [5]. The study is being conducted by the International Institute for Population Sciences (IIPS), Mumbai and in collaboration with the Harvard T H Chan School of Public Health and the University of Southern California. The total sample included 72,250 individuals aged 45 years and above and their spouses who reside in the same household irrespective of age. The response rate was 87.3% for the individual interview and 95.8% for the household interview. The study covers all Indian states and union territories except Sikkim. The information from respondents was collected through face-to-face interviews and self-administered questionnaires. The LASI adopted a multistage stratified area probability cluster sampling design to arrive at the eventual units of observation [5]. The data has been collected in such a way as to enable cross-state analyses and cross-national analyses of health, ageing, social behaviour, and economic status. Detailed information about the design of the study can be found in the LASI Wave-1 report [5].

### Study participants

LCA works only on complete cases of variables. Therefore, after excluding missing cases on the educational status of individuals, the analytical sample consists of 32,827 individuals aged 45 years and above. Individuals aged 45 years and above are included in the study.

In this study, four types of dependency domains, such as physical, social, psychological and economic, were used to identify the multidimensional dependency patterns. These four variables were available in the LASI wave-1 study, and therefore, a total of nine observed indicators were used to measure multidimensional dependency.

## Outcome variables and measurements

### Physical dimension

In this study, we used the Activities of Daily Living (ADL) and the Instrumental Activities of Daily Living (IADL) to measure the functional status of older adults. These two indexes are the most commonly used and globally accepted tools to measure the functional ability of older adults [7, 9]. ADL is a term used to refer to the ability to do basic daily self-care like moving from bed to chairs, dressing, bathing, walking across a room, eating, and use of a toilet. The ability or inability to perform these activities indicates the functional status of a person. Further, IADL is a scale that measures a person’s ability to live independently in a community based on tasks like preparing a hot meal, shopping for groceries, doing household work, making telephone calls, managing money, and getting around. Respondents were asked to respond “Yes” [1] or “No” (0) if they had difficulty performing these activities. The total sum score of these responses for each ADL and IADL was 0–6. In this study, we define dependency as those respondents reporting the inability to perform two or more out of six ADL activities. Similarly, we define a dependency for IADL as those respondents reporting the inability to perform two or more out of six IADL activities.

The other domain of physical limitation was also measured using the following activities: climbing a flight of stairs without resting and pushing or pulling a large object.

### Vision impairment

The eyesight quality of the respondents was measured using two self-reporting questions: reading eyesight and distance eyesight with or without wearing glasses, contact or corrective lenses.

### Social dimension

Several studies highlighted that widowhood is a situation where an individual may experience economic, social, and psychological problems [29–31] and the emotional stress resulting from widowhood may lead an individual to depression [29]. Widowhood was measured by the question, “What is your current marital status”. The response was dichotomised: currently married, divorced, separated, deserted, live-in-relationship, and never married as “2” and widowed as “1”.

### Psychological dimension

The depressive symptoms were assessed using the Centre for Epidemiologic Studies Depression (CES-D) scale [32]. The original CES-D scale is based on 20 items, while LASI adopted a shortened 10-item scale with four option categories [5]. The 10 items for assessing depressive symptoms include seven negative symptoms (trouble concentrating, feeling tired or low in energy, feeling depressed, afraid of something, feeling alone, bothered by things, and everything was an effort), three positive symptoms (feeling hopeful about the future, overall satisfied, and happy). The response for negative symptoms was dichotomised: rarely or never (less than 1 day), sometimes (1 or 2 days) as “0” and often (3 or 4 days) and most or all of the times (5 to 7 days) as “1”. We reverse-coded for the positive symptoms. The sum score of all 10 items ranged from 0 to 10, with scores 0–3 categorised as “2” (without depressive symptoms) and scores 4–10 categorised as “1” (with depressive symptoms).

### Cognitive dimension

The cognitive module of the Health and Retirement Study (HRS) was adopted in LASI to assess cognitive function. The various domains of cognitive measurement included memory (score 0–10), orientation (score 0–8), arithmetic (0–9), executive function (score 0–4), and object naming (score 0–2). The maximum score of the cognitive scale used is 43, and the respondent who scores at or below the 10th percentile point is categorised as having cognitive impairment [33].

### Economic dimension

Monthly per capita consumption expenditure (MPCE) was used to measure the household economic status. MPCE was measured using household consumption data. A list of 11 and 29 questions on the expenditure on food and non-food items respectively, was used to canvass the sample household [5]. MPCE was categorised as poorest, poorer, middle, richer, and richest. The poorest and poorer were categorised as “1” (economic dependence), and the middle, richer and richest were categorised as “2” (economic independence).

### Covariate or concomitant variables

**Fig. 1:**
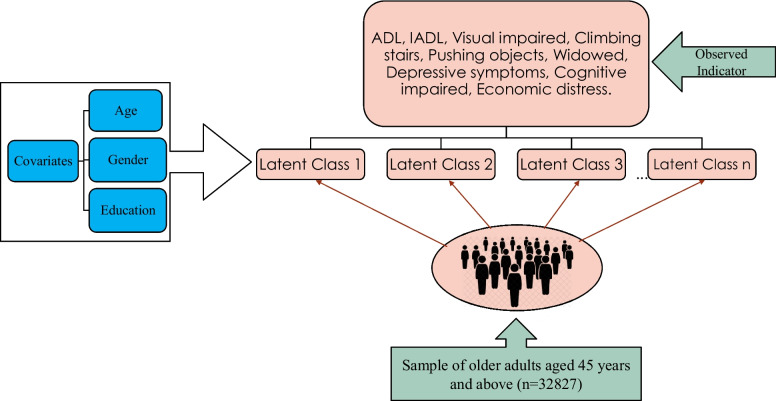
Conceptual framework of the study

Guided by the related literature, the covariate was selected for the analysis. The covariate variables used to predict the clustering of multidimensional dependency are demographic, social, and health behavioural risk factors. The demographic variables are age (45–49, 50–54, 55–59, 60–64, 65–69, 70–116), gender (male and female), and educational level (primary or below, upper primary, secondary, and above secondary). Figure 1 illustrates the conceptual framework of the study.

### Statistical analysis

In this study, LCA uses nine manifest dichotomous variables to identify the multidimensional dependency latent or unobserved sub-groups in a heterogeneous population of older adults. We used the latent class regression model to analyse the pattern of dependency of older adults. In this method, the outcome (dependency) is the category of latent class which is expressed in terms of observed manifest variables. The nine manifest variables measure the status of older adults in terms of daily activities, cognitive, and socioeconomic.

Supposed a latent class model with *C* classes is to be estimated based on the M polytomous categorical manifest variables (i.e., ADL, IADL, Physical domain, Vision impairment, Social dimension, Psychological dimension, Cognitive dimension, and Economic dimension), each of which contains *r*_*m*_ possible outcomes, and the covariates such as age, education, and gender. Let the vector *Y*_*i*_ = (*Y*_*i*1_, *Y*_*i*2_, …. . *Y*_*iM*_) represent the responses of individual *i’*s to the M manifest variables where *Y*_*im*_ =1,2,…. *r*_*m*_. Also, membership in the latent class for the *i*^*th*^ individual is denoted by *c*_*i*_ =1,2,….*C* and let the indicator function *I*(*y*_*im*_ = *k*) equal 1 if response *y* equals *k* and 0 otherwise. Let *ρ*_*mk* ∣ *c*_ is the class-conditional probability that a respondent in class *c* = 1,2…..C produces *k*^*th*^ outcomes on the *m*^*th*^ manifest variable, such that within each class, for each manifest variable $\sum_{k=1}^{r_m}{\rho}_{mk\mid c}=1$. Also, *γ*_*c*_ ($\sum_{c=1}^C{\gamma}_c$ =1) is the prior probability of membership in the latent class, and it represents the unconditional probability that an individual will be a member of each latent class before taking into account the response *y*_*im*_ provided by the manifest variable. If we incorporate the *p* × *1* vector of covariates or concomitant variables, ***x*** = (*x*_1_, *x*_2_, …. . *x*_*p*_)^′^ and its value is associated with the probability of membership in each latent class, *γ*. Then the mathematical model equation for LCA can be expressed as:

$$\textrm{P}\left(\textrm{Y}=y\left|\ x\right.\right)=\sum\nolimits_{c=1}^C{\gamma}_c\left(\boldsymbol{x}\right)\prod\nolimits_{m=1}^M\prod\nolimits_{k=1}^{r_m}{\rho}_{mk\mid c}^{I\left({y}_{im}=k\right)}$$

Where *m* = 1,2…..M represent the number of manifest variables and *k* = 1,2….. *r*_*m*_ represents the number of possible categories the variable *y*_*im*_ can take.

The prior probability that the individual has class membership *c* is given by the generalised-logit link function as

$${\gamma}_c\left(\boldsymbol{x}\right)=\frac{\exp \left\{{\boldsymbol{x}}^{\prime }{\boldsymbol{\beta}}_c\right\}}{\sum_{j=1}^C\exp \left\{{\boldsymbol{x}}^{\prime }{\boldsymbol{\beta}}_j\right\}}$$

Where ***β***_*c*_ = (*β*_1*c*_, *β*_2*c*_, …. . *β*_*pc*_)^′^ for c = 1,2,…..C-1 is a *p* × *1* vector of regression coefficients corresponding to the *c*^th^ class which influences the log odds that an individual belongs to the class *c* relative to the first class considered as the reference class. The estimated parameters from the latent class regression model are the *C*-1 vectors of coefficients ***β***_*c*_ and *ρ*_*mk* ∣ *c*_ the class-conditional probability without covariates. Given the estimates $\hat{\boldsymbol{\beta}}$_*c*_ and $\hat{\rho}$_*mk|c*_ of ***β***_*c*_ and *ρ*_*mk* ∣ *c*_, and *x*_*i*_ the observed covariates for the *i*^*th*^ individual, then the posterior class membership probabilities in the latent class regression model can be estimated using Bayes’s formula:

$$\hat{P}\ \left({c}_i\left|{x}_i;{Y}_i\right.\right)=\frac{\gamma_c\ \left({x}_i;\hat{\beta}\right)f\left({Y}_i;\widehat{\rho_{mk\mid c}}\right)}{\sum_{j=1}^C{\gamma}_j\ \left({x}_i;\hat{\beta}\right)f\left({Y}_i;\widehat{\rho_{mk\mid j}}\right)}$$

The number of latent classes is unknown; hence to determine the optimal number of latent classes, we examined one to six models. The latent class analysis involved exploring a range of potential latent class models, varying from one to six classes. To identify the most suitable number of latent classes, we employed a two-tiered strategy. In the first approach, we utilized widely recognized model selection criteria, including the Bayesian Information Criterion (BIC), traditional log-likelihood, and the likelihood ratio test. In the second approach, we applied a more comprehensive set of model selection criteria, incorporating the Lo-Mendell-Rubin (LMR) test and the Bootstrapped Likelihood Ratio (BLR) test statistics. In selecting the number of latent classes, we also took into consideration how well a class has theoretical meaning and could be easily interpreted [34]. To assess the association between the explanatory variables (i.e., age, gender, education) and the latent class, we adopted the one-step technique approach. In this approach, the explanatory variables are included in the latent class regression model, and their coefficient is estimated simultaneously as part of the latent class model [35]. This approach has been demonstrated to provide the best and most unbiased coefficient estimates of the explanatory variables as compared to other methods [36]. LCA was conducted in *R* statistical package [37], using the poLCA package (Polytomous Variable Latent Class Analysis) [35].

## Results

### Descriptive statistics

A total of 32,827 older adults aged 45 years and above were included in the analysis (Table 1). Individuals in the age group 45–49 and 65–69, respectively, constitute the largest (23.1%) and lowest (13%) proportion of the total sample. More than 40% of the sample respondents have an educational level up to the primary level, and more than 50% have completed at least a secondary level. Male respondent constitutes the largest (61.3%) proportion of the total sample, with females constituting only 38.8%. The proportion of respondents who reported being dependent for any ADL and IADL activities was 7 and 13%, respectively. More than 35% of the sample respondents reported facing difficulty in climbing a flight of stairs and pushing or pulling objects. Overall, a low proportion (3%) of older adults reported having symptoms of cognitive impairment, however, almost 35% reported facing economic distress.

**Table 1:** Percentage distribution of older adults by selected background characteristics in India, LASI (2017–18)

Background Characteristics	Sample	Percentage
**Age**		
45–49	7932	23.1
50–54	6126	18.3
55–59	5134	14.7
60–64	4904	14.8
65–69	4017	13.0
70–100	4714	16.1
**Education**		
Primary or below	15,092	45.9
Secondary or above	17,735	54.1
**Gender**		
Female	13,021	38.8
Male	19,806	61.3
**Activities of daily living (ADL)**		
Dependent	1930	7.0
Independent	30,897	93.0
**Instrumental activities of daily living (IADL)**		
Dependent	4154	12.7
Independent	28,673	87.4
**Climbing flight of stairs**		
Difficult	12,239	39.8
Not difficult	20,588	60.2
**Pushing or pulling objects**		
Difficult	10,614	35.1
Not difficult	22,213	64.9
**Marital status**		
Widowed	4645	15.3
Not Widowed	28,182	84.7
**Depression**		
With symptoms of depression	7183	23.8
No problem	25,644	76.2
**Cognitive**		
Suggestive cognitive impairment	978	3.0
No problem	31,849	97.0
**Economic condition**		
Suggestive of economic distress	10,714	34.8
No problem	22,133	65.3
**Total**	**32,827**	**100.0**

### Model fit and selection of latent classes

The model selection criteria, as presented in Table 2, guided our selection of the most plausible latent class model. We fitted latent class analysis (LCA) models, spanning from 1 to 6 classes, and for each of these models, we calculated several model fit indicators, including BIC, LMR, and BLR. These indicators collectively pointed to the four-class model as the most favourable option due to its combination of the lowest BIC value and the smallest likelihood ratio test statistic.

**Table 2:** Model fit indices of latent class analysis on multidimensional dependency

Model	Log-likelihood	BIC	Likelihood-ratio	LMR (p-value)	Log-BLRT (p-value)
**M1**	− 139,620.5	279,335	22,850.515	–	–
**M2**	−126,720.3	253,680	2717.820	M2 vs M1: 2499.06(*p* < 0.001)	17.44(*p* < 0.001)
**M3**	− 125,495.0	251,375	1278.601	M3 vs M2: 2374.56(*p* < 0.001)	18.22(*p* < 0.001)
**M4**	− 124,311.0	249,152	1386.040	M4 vs M3: 2294.38(*p* = 0.098)	18.08(*p* < 0.078)
**M5**	−126,314.6	253,305	2602.360	M5 vs M4: −0.0000(*p* = 1.000)	16.78(*p* = 0.878)
**M6**	−126,720.3	254,262	2097.516	M6 vs M5: − 4191.8(*p* = 1.000)	16.36(*p* = 1.000)

*LMR* Lo-Mendell-Rubin, *BLRT* Bootstrapped Likelihood Ratio Test

However, it’s worth noting that traditional likelihood ratio tests might not be as reliable when comparing two nested models with different class numbers [38]. Hence, we leaned on two additional fit indices, LMR and BLR. A notably low *p*-value when comparing M3 to M2 in Table 2 suggested that M3 is preferable to M2. Furthermore, the log of the BLR test statistic was highest for M3 (see Supplemental Fig. 2), and it accompanied the smallest *p*-value (see Supplemental Fig. 1).

**Fig. 2:**
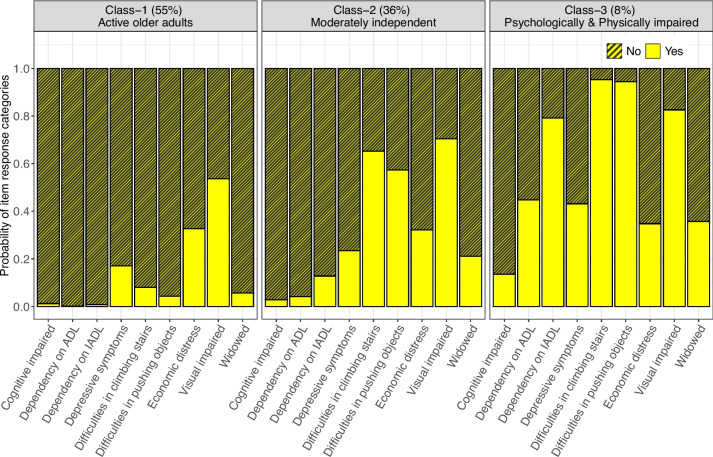
Item response conditional probabilities across the latent classes

The distributions of *p*-values and BLR test statistics across 100 bootstrapped samples for each fitted latent class are illustrated in graphs (see Supplemental Figs. 1 and 2). These visualizations indicated that the latent class model with three classes consistently yielded the highest number of classes among models with the smallest *p*-values, further reinforcing our choice of the three-class model as the most plausible selection. This decision was informed by a careful consideration of fit indices and the interpretability of the resulting classes [34]. The three latent classes Cluster 1, Cluster 2 and Cluster 3 were labelled as “Active older adults”, “Moderately independent”, and “Psychological and physically impaired”. The graphical representation of item response probability for the observed indicator’s variables across the latent classes is illustrated in Fig. 2.

Latent class probability and the conditional probability of a “Yes” response for each indicator variable are summarised in Table 3. The last row in Table 3 indicates the probability of class membership in each latent class. In Fig. 2, about 53% of the sample older adults were classified in the “Active older adults” class. Individuals in this class were characterised as having a very low probability of needing help for any ADL, IADL and other activities. Moreover, individuals in this class had a low probability of being widowed and cognitively impaired. Further, 37% of the sample older adults were classified to the other class, namely “Moderately independent”. Individuals assigned to this class are expected to need help with any basic IADL activities, climbing stairs and pushing objects. This class is also characterised as a class having a high probability of visually impaired individuals. Finally, the remaining 10% of the older adults were assigned to the class name “Psychological and physically impaired”, individuals in this class are expected to need help for any basic ADL, IADL, climbing stairs, and pushing objects. Also, they are most probably being visually impaired and depressed. Their expected probability of needing help with climbing stairs and pushing objects in this class is 0.9.

**Table 3:** The item response and class membership probabilities of the three latent classes

Observed indicators	Cluster 1: Active Older adults (***n*** = 17,697, 53%)	Cluster 2: Moderately independent (***n*** = 11,982, 37%)	Cluster 3: Psychological and Physically impaired (***n*** = 3148, 10%)
Dependency in ADL	0.001	0.042	0.448
Dependence in IADL	0.007	0.128	0.792
Visual impaired	0.536	0.705	0.826
Difficulties in climbing stairs	0.080	0.653	0.953
Difficulties in pushing objects	0.044	0.573	0.944
Widowed	0.056	0.211	0.357
Depressive symptoms	0.171	0.234	0.431
Cognitive impaired	0.012	0.028	0.135
Economic distress	0.326	0.321	0.347
**Class membership probability**	**0.55**	**0.36**	**0.08**

### Covariates predicting latent class membership

Table 4 summarises the findings from the latent regression model. Members in the “moderately independent” cluster were more likely to be comprised of older individuals (OR = 1.09; 95% CI: 1.08–1.10), who were less likely to complete a secondary level of education (OR = 0.68; 95% CI: 0.63–0.73), and less likely to be male (OR = 0.21; 95% CI: 0.13–0.36) as compared with members belonged to the “active older adults” cluster. Further, the “psychological and physically impaired” cluster comprised mostly of older individuals (OR = 1.19; 95% CI: 1.17–1.20), who were less likely to complete a secondary level of education (OR = 0.43; 95% CI: 0.38–0.47) as compared with members in the “active older adults” cluster. Also, female respondents were predominantly most likely to belong to this class.

**Table 4:** The odds for various latent classes by socioeconomic factors as revealed by the latent class regression model

Background Characteristics	Cluster 2: Moderately independent		Cluster 3: Psychological and Physically impaired	
	Odds Ratio	95% CI	Odds Ratio	95% CI
**Age**	1.09***	(1.08–1.10)	1.19***	(1.17–1.20)
**Education**				
Primary or below	**Ref**		**Ref**	
Secondary or above	0.68***	(0.63–0.73)	0.43***	(0.38–0.47)
**Gender**				
Female	**Ref**		**Ref**	
Male	0.21***	(0.13–0.36)	0.71***	(0.32–0.79)
Age*Gender	1.00	(0.99–1.01)	0.98***	(0.97–0.99)

Ref: Reference category; ***p-value< 0.01; CI: Confidence interval; Reference class: Cluster 1: Active older adults

**Fig. 3:**
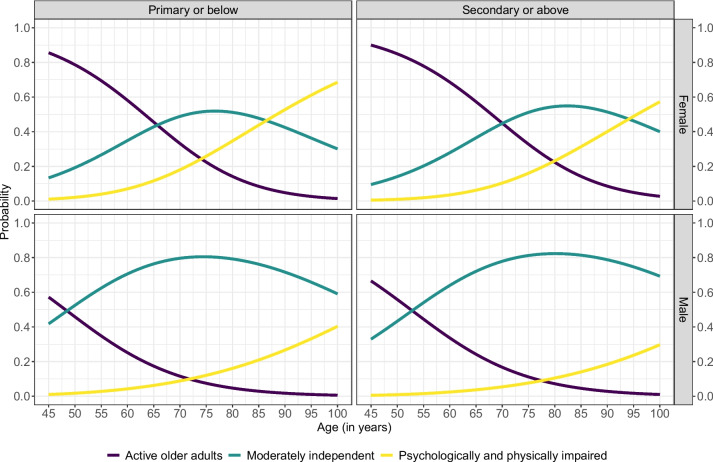
Probability of being assigned to each latent class by age, sex and level of education

Figure 3 depicts how the estimated probabilities of being assigned in each latent class vary with the respondent's age, classified by gender and educational attainment. The probability of being in the “Active older adults” class decreases with age, irrespective of gender and educational attainment. We observed a sudden decrease in this probability for individuals aged 50 years or older. Further, the results indicate gender differences in the probability of being assigned to each of the three latent dependency classes. For example, the probability of being assigned to a “Psychological and physically impaired” class is almost zero for men under the age of 60 years for both levels of educational attainment. However, these probabilities are higher for females of at least 60 years old and even higher for those with primary or lower levels of education. Moreover, the probability of being assigned to the “Active older adults” class is higher among younger females with higher educational attainment.

## Discussion

The objective of this study was to identify the characteristics of older adults from India by their multidimensional dependency pattern using a latent class approach. We identified three heterogeneous groups of dependent older adults and analysed the profile of these groups and the socio-demographic profile of these groups. Based on the nine indicator variables, the three classes that were identified were “Active Older adults”, “Moderately independent”, and “Psychological and physically impaired”. The profiles of the “Active older adults” consist of the majority of individuals who are unlikely to need help for any ADL, IADL, or physical activities. The “Moderately independent” profile comprised the individuals who exhibited difficulties in climbing stairs, pushing objects and being visually impaired. Finally, the “Psychological and physically impaired” profile, the smallest subgroup, comprised of individuals who exhibited a high dependency profile, that is the members of this group will most probably need help for any basic ADL and IADL, will require help to climb the stairs and push objects. In line with the previous findings, our results support that the older population exhibit a different pattern of ageing, which leads to heterogeneity in health conditions, dependency, and disability [13, 39–41].

Further, we assessed the role of age, education and gender on the probability of belonging to any of the three dependency classes, and the results indicate that the probabilities vary according to the socio-demographic characteristics. We found that the members in the “Moderately independent” and “Psychological and physically impaired” classes were comprised mainly of individuals of older age, with low levels of schooling as compared to the members in the “Active older adults” class. Also, being female significantly increased the risk of being a member of the “Moderately independent”. Members of the “Psychological and physically impaired” group have a high probability of being widowed, physically impaired, psychologically impaired, and suffering from economic distress. In agreement with the previous study, the prevalence of dependency was very high in this group, and the members were predominantly female of older age [14]. This evidence is consistent with the “male-female survival health paradox” since females live longer than males and are more likely to depend on others for any ADL, IADL activities [42]. Further, gender discrimination against females in a male-dominated society like India makes females more vulnerable to the risk of poor health and disability [43].

It is well documented that the heterogeneous groups of older age with different health profiles consume a disproportionate and inappropriate share of health care services [44]. This study also shows that individuals in the “Psychological and physically impaired” group report more functional and economic problems than the individuals in the “Active older adults” and “Moderately independent” group. It is obvious from the results of this study that the prevalence of dependency is very high in this group. The probable reason behind this phenomenon is the advanced age and waning body in the final part of life. Older individuals with inadequate economic/financial resources will be limited from accessing health care services and nursing homes, and this will result in not being able to fulfil their needs of daily life properly [44, 45]. This approach of examining the effect of heterogeneity of dependency and exploring their socio-demographic characteristics will be helpful for the policy and decision maker to understand the health and dependency status of older adults, to plan effective care or services that cater for the needs of the older adults and to devise a plan for effective allocation of resources.

To our knowledge, this is the first study in India that used the latent class approach to examine the multidimensional dependency status of older adults based on the dependency domain, such as physical, psychological, economic, and social dependency. The principal idea of latent class analysis is to identify the unobserved latent categorical variable that describes the relationship among the set of observed indicators. Latent class analysis has two advantages. First, the misclassification error is minimised because we don’t have to assign older adults to a specified class, as the model has assigned each older adult an estimated probability of class membership. Secondly, the latent class regression model, allows us to simultaneously incorporate the covariates variables other than the observed variables in assessing their independent association with the dependency latent class.

## Conclusions

In summary, this study identified three separate, broad dependency profiles using the latent class analysis in an Indian nationally representative sample of older adults. These three groups have distinct physical, psychological, economic, and socio-demographic characteristics in terms of activities in which help is required. It has been acknowledged that multidimensional dependency has been associated with a greater burden and increased use of health care. This finding is key information to the planning and designing of care services and the broadening of assistance to the older population, especially in developing countries with limited resources. Future research should focus on the mechanism of these multidimensional dependencies and explore strategies for prevention and intervention.

### Supplementary Information

**Supplementary Material 1.**

**Supplementary Material 2.**

## Data Availability

The dataset supporting the conclusions of this article is publicly available in the Gateway to Global Aging Data, https://g2aging.org/.

## Abbreviations

IIPS: International Institute for Population Sciences
LASI: Longitudinal Ageing Study in India
ADL: Activity of Daily Living
IADL: Instrumental Activities of Daily Living
LCA: Latent Class Analysis
BIC: Bayesian-Schwarz Information Criterion
WHO: World Health Organisation
OR: Odds Ratio
SPM: Strong P Marbaniang
HSC: Holendro Singh Chungkham
