# Supplementary material for: Latent class of multidimensional dependency in community-dwelling older adults: evidence from the longitudinal ageing study in India
Source: BMC Geriatr. 2024 Feb 28;24:203. doi: 10.1186/s12877-024-04813-9 (PMC10900629; doi:10.1186/s12877-024-04813-9)
Supplement: Supplementary file 1 — Supplementary Material 1. [file 12877_2024_4813_MOESM1_ESM.docx]

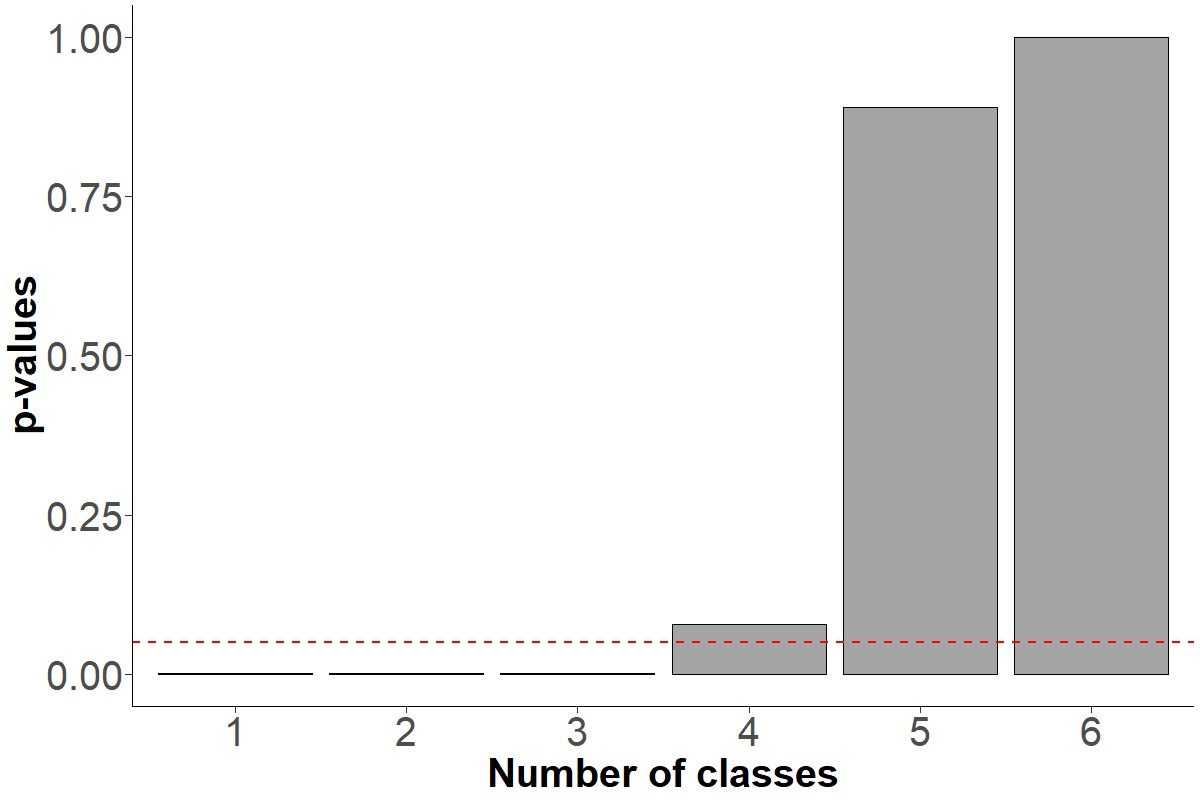


Supplemental Figure 1: Distribution of *p-*values of bootstrapped likelihood ratios for competing latent classes. The horizontal dashed line indicates the *p-*value at 5%.


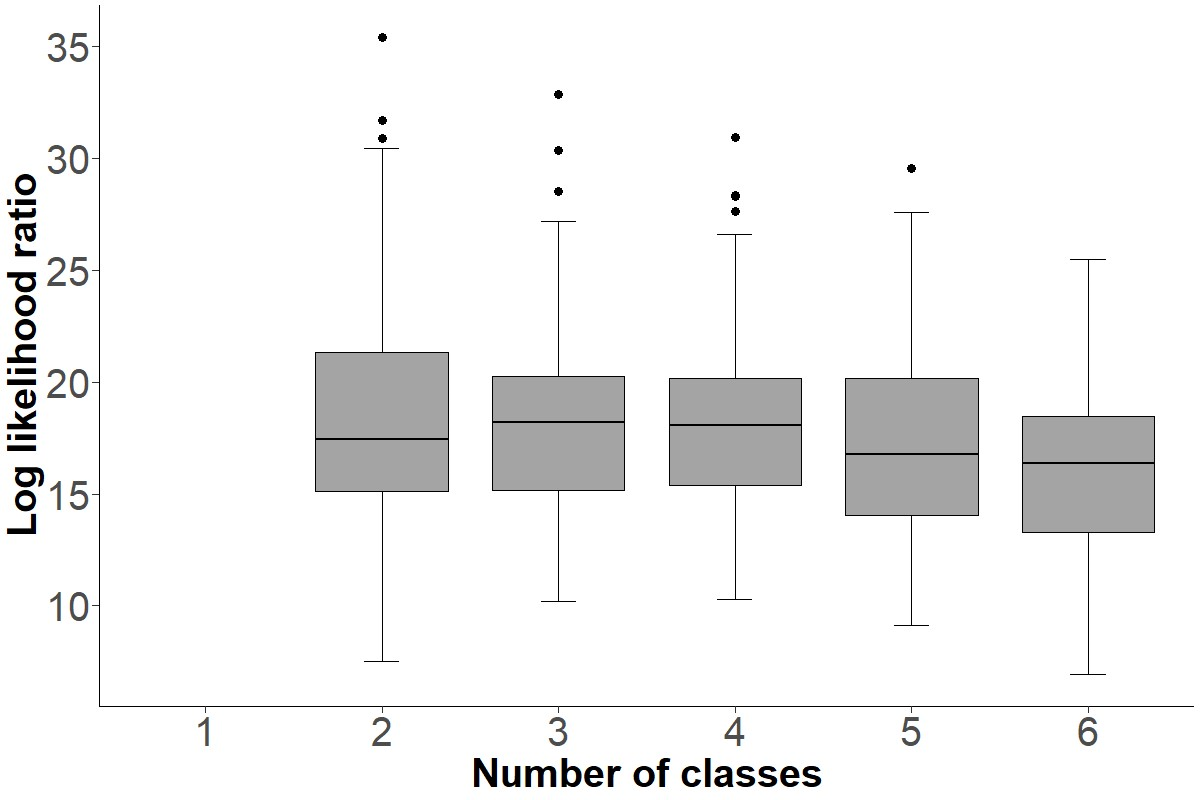
Supplemental Figure 2: Distribution of bootstrapped likelihood ratios for competing latent classes.
